# Impact of contact lens correction on wavefront aberrations and vision quality in keratoconus

**DOI:** 10.1111/opo.70037

**Published:** 2025-10-29

**Authors:** Gavin Swartz, Khyber Alam, Alex Gentle, Laura E. Downie

**Affiliations:** ^1^ Department of Optometry and Vision Sciences School of Health and Clinical Sciences, the University of Western Australia Perth Western Australia Australia; ^2^ School of Medicine, Faculty of Health Deakin University Geelong Victoria Australia; ^3^ Department of Optometry and Vision Sciences, Faculty of Medicine, Dentistry and Health Sciences The University of Melbourne Melbourne Victoria Australia

**Keywords:** contact lens, higher order aberrations, keratoconus, RGP, scleral lens

## Abstract

**Purpose:**

To review the optical principles of contact lens correction in keratoconus, with a focus on residual ocular wavefront error and its impact on vision performance.

**Findings:**

Corneal rigid gas permeable (RGP) contact lenses, commonly used in keratoconus management, often fail to improve vision quality to levels observed in non‐diseased eyes due to residual higher‐order aberrations (HOAs), primarily originating from posterior corneal irregularity. Dependent on the nature of the residual wavefront error, various lens parameter modifications, including optic zone asphericity, base‐down prism and scleral lens vault adjustments, can improve vision outcomes. Wavefront‐guided corrections have demonstrated potential to provide vision quality equivalent to that of healthy eyes, particularly when sufficient time is allowed for neural adaptation.

**Summary:**

This review examines the optical characteristics of the cornea in keratoconus, including the induction of elevated ocular wavefront errors. Challenges relating to optical correction with contact lenses are discussed, with an exploration of the origins of residual HOAs and their subsequent impacts on vision quality and quality‐of‐life. A range of contact lens modalities and parameter modifications are discussed, with evidence supporting their efficacy in reducing residual ocular wavefront error. Emerging data on wavefront‐guided corrections are presented, along with future directions for personalised optical corrections tailored to individual patient presentations.

**Key points**

- Individuals with keratoconus may experience reduced vision quality and quality‐of‐life due to persistent optical distortions, even with use of rigid contact lenses.
- The posterior tear lens formed by a rigid contact lens corrects for corneal surface irregularities but does not address internal distortions, which continue to degrade visual performance.
- Modifications to the contact lens design, including personalised approaches targeting internal distortions, may offer superior vision outcomes compared to conventional rigid lenses in keratoconus management.

## INTRODUCTION

Keratoconus, the most common primary corneal ectasia, is characterised by bilateral thinning of the cornea with associated asymmetric topographic alterations that can ultimately lead to vision impairment.^1^, ^2^, ^3^ Although the global prevalence of keratoconus is estimated at 1.38 per 1000 in the overall population,^2^ with significant regional variation^1^, ^2^, ^4^, ^5^ higher figures have been reported, including a prevalence of 1 in 84 in Western Australia.^4^ With onset often during the late teenage years,^1^ keratoconus impacts visual function significantly for many individuals during their secondary and tertiary education years,^6^ and can lead to activity limitations that impact working opportunities and quality of life (QoL).^7^

The increasing vision impairment associated with keratoconus progression results from optical distortions induced by the increasingly asymmetric corneal profile.^1^, ^8^ These higher‐order aberrations (HOAs), including spherical aberration (denoted by the Zernike polynomial term $Z_{4}^{0}$) and coma ($Z_{3}^{-1},Z_{3}^{1}$), are not correctable with standard refractive correction modalities like spectacles and soft contact lenses. Instead, more specialised approaches are often required, with rigid gas permeable (RGP) contact lenses the mainstay.^1^, ^9^, ^10^ While these rigid contact lenses improve visual function by significantly reducing HOAs,^11^, ^12^ the presence of residual HOAs often limits the vision level achieved and can adversely impact the QoL.^7^

Despite the significant advancements in contact lenses for keratoconus in recent years, the persistence of residual HOAs following correction remains a challenge for many, limiting their overall success with this vision correction modality. This review examines evidence pertaining to the origins and implications of these residual HOAs, synthesising evidence around current management strategies, including wavefront‐guided corrections, while identifying future directions for a more targeted approach based on individual presentations.

### HOAs and vision in keratoconus

In keratoconus, alterations of the cornea's biomechanical properties result in central and paracentral stromal thinning, ultimately leading to deformation of both the anterior and posterior corneal surfaces.^13^ The anterior interface of the cornea with air represents the largest change in refractive index between the ocular structures and media involved in image formation, making it the eye's most powerful optical element.^14^ As a result, the increasing departure from rotational symmetry of the cornea during keratoconus progression leads to increasingly negative impacts on image quality. Central steepening induces myopic defocus, with spherical aberration ($Z_{4}^{0}$) becoming less positive (or more negative), while the (commonly encountered) inferior displacement of the cone additionally leads to increased magnitudes of astigmatic error and asymmetric HOAs.^13^, ^15^, ^16^, ^17^, ^18^, ^19^, ^20^, ^21^, ^22^, ^23^, ^24^, ^25^ Dominated by vertical coma ($Z_{3}^{-1}$), the magnitude of the root mean squared error (RMS) for ocular HOAs in total is commonly more than six times that of non‐diseased eyes,^21^, ^23^, ^26^ and increases with disease severity.

A linear relationship is observed between higher‐order root mean squared error (HORMS) and image quality, with degradation of the retinal image rising in‐step with elevations in the magnitude of HOAs.^22^, ^24^, ^27^, ^28^ Although exacerbated with larger pupils,^29^ apodisation imparted by the Styles‐Crawford effect^30^, ^31^ means Zernike terms with greater influence on the central ocular wavefront are more visually impactful than those that act more peripherally.^20^, ^27^, ^28^, ^32^, ^33^ For this reason, it is commonplace for researchers to report on HOAs of low angular frequency like spherical aberration ($Z_{4}^{0}$) and coma ($Z_{3}^{-1},Z_{3}^{1}$) when considering image quality in keratoconus.^21^, ^24^

The retinal image is formed by the entire ocular wavefront, the shape of which is described by the sum of its component lower‐ and HOAs.^32^, ^33^, ^34^, ^35^, ^36^, ^37^, ^38^ Limitations imparted by neural processing constrain what detail within the wavefront may be perceived.^29^, ^39^, ^40^, ^41^ Therefore, one cannot consider the impacts on vision quality for the RMS of an individual aberration term in isolation. Instead, quality metrics such as the Visual Strehl ratio (VSX) are required.^36^ This metric quantifies the reduction in the contrast of a point spread function (PSF) aberrated by the ocular wavefront, relative to its un‐aberrated counterpart, while accounting for limitations imposed by neural processing in both.^36^, ^40^, ^42^

### Vision correction in keratoconus

In the early stages of keratoconus, the ocular wavefront remains dominated by defocus ($Z_{2}^{0}$) and astigmatism ($Z_{2}^{-1}$, $Z_{2}^{1}$),^17^ both lower order aberrations (LOAs) and correctable by standard spherocylindrical optics.^43^ Spectacles and soft contact lenses are therefore viable options for vision correction,^3^, ^8^, ^13^, ^44^, ^45^, ^46^, ^47^ although, with these modalities, HOAs remain higher and VSX lower than in healthy eyes.^45^, ^48^ As HORMS increase with keratoconus progression, these vision correction modalities become increasingly less suitable. Rigid contact lenses, including corneal, corneoscleral, hybrid and scleral lenses, are often the main mode of vision correction for individuals with keratoconus, as they offer a significant reduction in HORMS,^13^, ^25^, ^26^, ^29^, ^41^, ^47^, ^48^, ^49^, ^50^ affording substantial improvement in visual acuity and binocular function compared to spectacles.^51^, ^52^, ^53^, ^54^, ^55^

Morgan and Efron conducted a global survey‐based longitudinal trend analysis of new contact lens fittings performed by practitioners between 2000 and 2020, and noted a resurgence in the use of RGPs; they attributed this, at least in part, to recent technological innovations in this modality.^56^, ^57^ It is estimated that 90% of individuals with keratoconus utilise rigid contact lenses for vision correction.^1^ Scanzera et al. analysed changes in contact lens prescribing for keratoconus at a US‐based tertiary facility between 2010 and 2020, noting an increase in the use of scleral lenses to 22% of cases compared to a baseline of zero a decade earlier. Although corneal RGPs remained the most common modality, prescribed in 60% of cases, this accounted for a 9% decline over the period. Additionally, the use of soft and hybrid lenses declined by 5% and 8%, respectively,^58^ further highlighting the surge in popularity of scleral lenses in keratoconus,^57^, ^59^, ^60^, ^61^ (Table 1).

**TABLE 1:** Standard vision correction modalities in keratoconus.

Modality	Indications	Clinical implications	Advantages	Disadvantages	Special considerations
Spectacles	Mild keratoconus^a^ Low HORMS magnitude relative to LOAs	Provide partial reduction of the ocular wavefront error through correction of LOAs Aniseikonia if an anisometropic prescription is indicated Less efficacy with disease progression	Non‐invasive Low‐cost Widely available Ease of use Range of lens designs available with ability to tailor to task‐related working distances	HOAs left uncorrected → Inadequate vision quality for moderate to severe cases Potentially thicker/heavy lenses with higher myopic astigmatism	Astigmatism magnitude increases with increasing displacement of the cone from the visual axis^17^ Visual comfort with neural adaptation Suitability based on task dependent vision requirements May be suitable for backup or emergency pair
Standard soft contact lenses	Mild keratoconus^a^ Low HORMS magnitude relative to LOAs	Provide partial reduction of the ocular wavefront error through correction of LOAs Less efficacy with disease progression	Widely available Simple to use No spectacle magnification Range of modalities Strong comfort profile	Minimal correction of HOAs → Inadequate vision quality for moderate to severe cases^a^ and possible dysphotopsia Instability of vision associated with lens rotation for spherocylindrical corrections	Best performance with central cone location High astigmatism requires good rotational stability Disposable lens options restricted by available parameters Thicker lenses may partially mask corneal surface irregularity^8^
Corneal RGPs	Appropriate for all disease stages	Provide significant correction of the ocular wavefront error with correction of LOAs and reduced HORMS Significant improvements in vision performance	Typically better vision performance than spectacles or standard soft contact lenses Highly customisable Ease of lens application and removal Tear exchange profile supports corneal physiology Broad practitioner familiarity	Residual HOAs Decentration and movement results in variability in vision Reduced initial comfort (period of adaptation required) Poor performance in dusty environments Poor stability with extreme or rapid ocular excursions → increased risk of lens ejection from the eye	A wide variety of lens designs are available to suit different cone morphologies Additional optical corrections available—aspheric and front surface toric corrections Severe keratoconus^a^ and decentred cones more challenging to achieve good centration Significant practitioner skill required
Hybrid contact lenses	Mild to moderate keratoconus^a^	Provide significant correction of the ocular wavefront error with correction of LOAs and reduced HORMS through rigid lens optics	Good comfort Good centration and stability Good performance in dusty environments	Residual HOAs Require more frequent replacement than rigid lens options Insertion and removal more challenging Limited availability More challenging to fit sagging cones and severe disease^a^	Provide comfort, stability and centration via soft skirt Limited customisation of lens optics—residual astigmatism left uncorrected where a toric front‐surface is unavailable Designs with low‐Dk hydrogel skirt limit wear‐time due to induced corneal hypoxia Regular replacement results increased costs
Scleral contact lenses	Potentially appropriate for all disease stages^a^	Provide significant correction of the ocular wavefront error with correction of LOAs and reduced HORMS Significant improvements in visual performance	Typically, better visual performance than standard vision correction modalities Highly customisable Good comfort Good/consistent centration Good rotational and translational stability Good performance in dusty environments	Residual HOAs Practitioner expertise required → less availability than other lens modalities Potentially more challenging lens application and removal Higher cost than other lens modalities	Corneal vault means lens design parameters are not constrained by cone morphology or disease severity^a^ Consistent lens centration and stability afford additional customisation of optical correction Low Dk materials or excessive clearance can result in corneal oedema due to hypoxia

Abbreviations: Dk, constant of diffusion (material oxygen permeability); HOAs, higher‐order aberrations; HORMS, higher‐order root‐mean‐squared error; LOAs, lower order aberrations; RGPs, rigid gas permeable contact lenses.

^a^ Based on the keratometric grading scale.

## OPTICAL PRINCIPLES: RIGID CONTACT LENSES AND OCULAR WAVEFRONT ABERRATIONS

Unlike soft contact lenses, which transfer any irregularities on the ocular surface through to their anterior surface, a corneal RGP lens retains its prescribed shape when placed on the eye.^41^ With specific lens parameters typically based on clinical presentation and the practitioner's fitting philosophy,^1^, ^10^, ^43^ generally the posterior surface is designed to partially emulate the corneal contour to ensure a lens design that optimises stability and centration while allowing for sufficient post‐lens tear exchange. Lens movement and tear exchange are required to maintain appropriate corneal physiology, with approximate rather than ‘perfect’ alignment desired. The tear film fills the space created between the lens and the anterior ocular surface, forming an additional optical element often referred to as the ‘tear lens’, with surfaces mirroring those that it abuts.^8^, ^9^, ^62^, ^63^, ^64^

With minimal disparity in refractive index between the tear lens (*n*_tears_ = 1.337) and neighbouring cornea (*n*_cornea_ = 1.376), the posterior tear lens interface neutralises approximately 90% of optical power arising from the anterior cornea,^57^, ^64^, ^65^ including HOAs.^1^, ^8^, ^9^, ^10^, ^13^, ^15^, ^25^, ^43^, ^48^, ^49^, ^64^, ^65^, ^66^, ^67^, ^68^, ^69^, ^70^, ^71^, ^72^ With a larger difference in refractive index from the RGP material, the anterior interface of the tear lens imparts spherocylindrical power, with curvature defined by the rigid contact lens' back optic zone radius (BOZR). Modifications to this parameter, often used for fitting adjustments, induce changes in tear lens back vertex power (BVP). The bulk of a rigid lens' spherocylindrical power is generated at the anterior surface, with its curvature designated as the front optic zone radius (FOZR).^9^, ^11^, ^12^, ^68^, ^69^, ^70^, ^72^ This parameter can be used to adjust the lens BVP, allowing for correction of refractive error that remains after accounting for the influence of the tear lens.

The optical surfaces of rigid contact lenses are typically more curved than those of spectacle lenses. When observed in air, a rigid lens with standard spherical or spherocylindrical surfaces imparts significantly more spherical aberration ($Z_{4}^{0}$) than a spectacle lens of the same BVP.^9^, ^31^, ^62^, ^73^ However, once placed on the eye, the tear lens neutralises a large portion of the spherical aberration ($Z_{4}^{0}$) arising from the posterior surface of the rigid contact lens, plus around 90% arising from the anterior cornea. Aberrations of the anterior lens surface remain; however, in the case of low BVPs this curvature is close to the anterior cornea, resulting in minimal change to ocular spherical aberration ($Z_{4}^{0}$) compared to a spectacle correction. When high BVPs are required, the necessary FOZR deviates from the corneal profile, inducing changes in spherical aberration ($Z_{4}^{0}$), with increases for positive powers and negative shifts for minus powers.^31^, ^62^, ^64^, ^74^ While adverse impacts associated with lens‐induced spherical aberration ($Z_{4}^{0}$) can be offset by aspheric surfaces within the optic zone,^9^, ^74^ with a significantly larger change in refractive index at this interface with air, front‐surface asphericity is more impactful.^73^

Displacement of the optic zone of rigid lenses relative to the visual axis induces both defocus ($Z_{2}^{0}$) and coma ($Z_{3}^{-1}$, $Z_{3}^{1}$), increasing in magnitude with the extent of decentration, especially when aspheric surfaces are present.^9^, ^29^, ^70^, ^73^, ^74^, ^75^, ^76^ Ray‐tracing simulations from 20 healthy (normal) eyes and 20 keratoconic synthetic schematic eyes were employed by Rozema et al. to model changes in vision quality associated with corneal RGP lens decentration and rotation. Eyes with keratoconus were more sensitive to deviations from the ideal position, with clinically significant changes in vision (defined here as a loss of more than two letters of logMAR acuity derived from VSX) observed for decentration as low as 0.27 ± 0.13 mm and rotation of 10.6° ± 4.8° in this sample.^67^ This can have implications for the image quality achieved during contact lens wear, considering the influence of the inferiorly displaced corneal apex in keratoconus. Fluctuations in image quality arise due to the dynamic nature of the location of a corneal RGP lens on the cornea during wear; with vertical translations up to 2 mm and rotations as high as 15°.^1^, ^44^, ^67^, ^71^, ^73^ Where centration and stability are challenging to achieve, clinicians should weigh the benefits to vision of using aspheric surfaces to offset high levels of spherical aberration ($Z_{4}^{0}$) against the additional coma ($Z_{3}^{-1}$, $Z_{3}^{1}$) induced by any (inadvertent) misalignment.

## RIGID CONTACT LENS CORRECTION OF KERATOCONUS

Despite the significant improvements in visual acuity afforded by rigid contact lenses for individuals with keratoconus, visual performance metrics typically fail to reach equivalent age‐matched normative levels.^13^, ^41^, ^51^ Many authors report a 60%–70% reduction in HORMS with the use of rigid contact lenses in highly aberrated eyes,^11^, ^13^, ^25^, ^26^, ^48^, ^66^, ^67^, ^76^, ^77^, ^78^ with the magnitude of uncorrected HOAs remaining elevated compared to healthy corneas. The origins of these residual HOAs are multifactorial,^49^, ^51^, ^64^, ^66^, ^77^, ^78^ and account for the reduced vision quality experienced in keratoconus despite correction with rigid contact lenses.

### Residual HOAs

The mismatch of refractive index between the tear lens and cornea means around 10% of HOAs arising from the anterior corneal interface are left uncorrected when a rigid lens is placed on the eye.^64^, ^65^, ^74^, ^78^ While insignificant in the case of regular corneas, where HORMS are low,^79^ this contributes to a portion of the residual aberrations; this is particularly relevant in advanced keratoconus, where anterior corneal irregularity is pronounced.^20^, ^23^, ^24^

As the tear lens created by a rigid contact lens has no influence at the cornea's posterior surface, any HOAs generated at this interface with the aqueous humour remain uncorrected.^1^, ^11^, ^15^, ^25^, ^41^, ^62^, ^64^, ^78^ With a smaller change in refractive index than the anterior interface with air, HOAs that arise from the posterior cornea of non‐diseased eyes are around one third in magnitude. In addition, as the shift in magnitude of refractive index is the reverse of the anterior interface, these HOAs are mostly opposite in sign, imparting a partial compensatory effect; therefore, total corneal HORMS are lower than those arising from its anterior surface.^11^, ^80^

Although anterior corneal irregularity is elevated in advanced keratoconus, so too is irregularity of the posterior cornea.^1^, ^11^, ^15^, ^19^, ^21^, ^24^ In a study that included 82 eyes with keratoconus, Chen and Yoon observed average HORMS of the posterior surface for a 6 mm pupil increased from 0.19 ± 0.05 μm in healthy corneas to 0.24 ± 0.05, 0.54 ± 0.21 and 1.04 ± 0.31 μm in mild, moderate and advanced keratoconus, respectively. While all posterior surface HOAs were elevated relative to non‐diseased corneas, coma ($Z_{3}^{-1}$, $Z_{3}^{1}$) remained dominant, whereby the average vector magnitude for advanced disease increased by more than 10‐fold to 0.93 ± 0.35 μm. Secondary astigmatism ($Z_{4}^{-2}$, $Z_{4}^{2}$), trefoil ($Z_{3}^{-3}$, $Z_{3}^{3}$), spherical aberration ($Z_{4}^{0}$) and quadrafoil ($Z_{4}^{-4}$, $Z_{4}^{4}$) were elevated by approximately 5.75×, 2.5×, 2× and 1.7×, respectively.^11^

**FIGURE 1:**
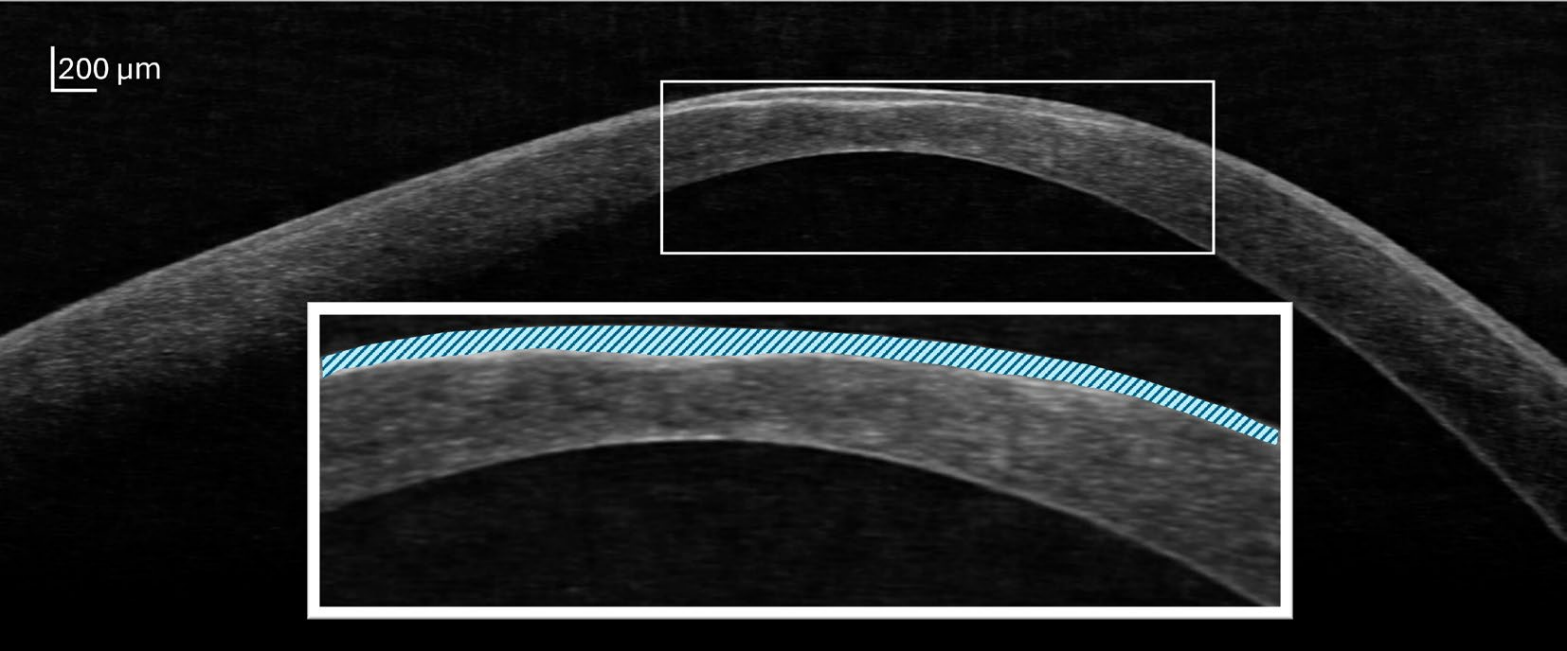
Corneal epithelial thickness variations in keratoconus. OCT B‐scan demonstrating regional epithelial thickness variation in a cornea with advanced keratoconus. The inset displays a magnified image of the area contained within the white box on the main image; light blue textured shading highlights the corneal epithelium. Here the epithelium is observed to increase in thickness overlying areas of stromal thinning, imparting a smoothing influence on the anterior corneal profile. In contrast, the curvature at the cornea's posterior interface is observed to be significantly steeper.

Variations in epithelial thickness in regions of stromal ectasia impart a smoothing influence on the ocular surface, partially masking anterior corneal irregularity.^81^, ^82^ Although dampening the HORMS arising from the cornea's external interface, the epithelium has no smoothing influence on the internal surface; therefore, the posterior cornea provides compensation for the anterior corneal HORMS to a greater degree in eyes with keratoconus than in healthy eyes. (Figure 1) While the anterior cornea remains the largest contributor to total corneal HORMS, rigid contact lens correction leaves the significantly elevated internal irregularity uncorrected, with this having the largest contribution to residual HORMS.^1^, ^11^, ^12^, ^15^, ^25^, ^48^, ^66^, ^68^, ^70^

Akin to the posterior cornea, HOAs arising from the crystalline lens partially compensate ocular aberrations.^38^, ^80^, ^83^, ^84^ These are also not corrected by rigid contact lenses, and thus contribute to residual HOAs, although to a lesser extent.^15^, ^62^, ^64^, ^74^, ^78^ Furthermore, other contact lens‐related factors contribute to elevated HORMS, including decentration, rotation, tilt and flexure, as well as the optical properties of the lens material, and poor surface wetting.^12^, ^15^, ^29^, ^64^, ^74^, ^76^

#### Influence on vision

The residual HOAs experienced during rigid contact lens wear in keratoconus can degrade retinal image quality, which limits their potential vision benefit. Considering that the asymmetric HOAs comprising the ocular wavefront have varied influences on the PSF,^17^, ^85^ HORMS alone does not capture image quality reliably.^28^, ^34^, ^41^ Instead, the modulation transfer function (MTF), derived from a Fourier transform of the PSF, affords additional insight, plotting maximal achievable contrast of an optical system across various spatial frequencies.^85^, ^86^ Using ocular wavefront data from 19 eyes with keratoconus, Pantanelli et al. calculated the change in MTF for full correction of HOAs, yielding on average 4.4 ± 2.0 times improved image quality compared to standard spherocylindrical refractive correction.^63^ In a similar study, Guirao et al. demonstrated as much as 25 times improvement for one individual.^85^ This disparity highlights the heterogeneity of keratoconus, where variations in corneal morphology and disease severity can induce marked variance in image quality. Although both studies examined the influence of HOAs on image quality with standard spherocylindrical correction, this holds true for the influence of residual HOAs when rigid contact lenses are used for vision correction.

In only considering image quality without the influence of neural processing, the MTF remains a poor predictor of a person's overall visual function. However, the VSX considers the visual system's ability to resolve detail in the setting of degraded image quality.^39^ Although underestimating vision performance in highly aberrated eyes,^40^ the change in logVSX observed when HOAs are corrected accounts for 80% of the variance in changes to logMAR visual acuity,^87^ and may therefore be useful in predicting potential improvements in vision associated with the correction of residual aberrations.

In standard spherocylindrical refractive correction of keratoconus, elevated asymmetric aberrations, including vertical coma ($Z_{3}^{-1}$), result in the subjective perception of inferiorly‐oriented, elongated tails and ghosting on images.^26^, ^70^, ^88^ Where habitual corrections provide chronically reduced image quality, a person's neural sensitivity can (automatically) adjust with a goal to enhance overall visual performance, including to reduce awareness of dysphotopsia.^26^, ^39^, ^49^, ^89^ With rigid contact lens correction, the dominance of anterior corneal aberrations is replaced by those arising from the internal surface of the cornea, with opposing orientation^11^, ^70^, ^80^; although reduced in magnitude, this manifests as a reversal in the direction of the prior image distortion.^26^, ^70^ As a result, despite an improved image quality, the alteration of the ocular wavefront from that which has informed a person's neurally‐adapted state means that many neophyte wearers of rigid contact lenses subjectively report poorer overall vision performance (relative to their habitual correction), with an increased perception of image distortion.^13^, ^41^, ^77^, ^88^, ^89^, ^90^, ^91^ Adaptation to the altered state induced by rigid lens wear does occur; however, the time course of this adaptation is dependent on the magnitude of change, as well as the duration of the individual's prior form of refractive correction.^15^, ^91^, ^92^

Interocular discrepancies in disease severity and the varied regional asymmetry of the cornea commonly observed in individuals with keratoconus can adversely impact binocular performance compared to those with non‐diseased eyes. As binocular summation and stereopsis rely on interocular retinal correspondence and disparity, respectively,^53^, ^54^, ^93^ the differences in regional contrast and phase shifts that result from asymmetric ocular wavefronts^54^ can lead to increased perceptual suppression of the input from the eye with poorer image quality,^52^ adversely impacting stereopsis. However, the reduced residual wavefront error provided by rigid contact lens correction, compared to spectacles, works to limit interocular conflicts and can significantly enhance stereopsis.^51^, ^52^, ^53^, ^54^, ^55^ Kumar et al. demonstrated this across a spectrum of keratoconus severities, with rigid contact lenses providing improved visual performance by up to 0.70 logMAR for high‐contrast visual acuity and accompanied by 10‐fold enhancements in stereoacuity in advanced keratoconus.^51^ In agreement with these findings for rigid lens correction compared to spectacles, Nilagiri et al. observed a similar trend in both unilateral and bilateral keratoconus, showing the benefits of equalising visual quality for best binocular function; however, in the presence of residual HOAs, neither cohort in this study achieved equivalent visual acuity or stereoacuity of those with healthy eyes.^53^

#### Quality of life

Although quality of life (QoL) assessment tools are not routinely employed in eye care practice,^59^ the Keratoconus Outcome Research Questionnaire (KORQ) is a keratoconus‐specific validated survey instrument designed to quantify vision‐related QoL, exploring the subdomains of symptoms and activity limitation.^7^, ^94^, ^95^ Utilising this instrument, Gothwal et al. demonstrated an association between elevated HOAs in advanced keratoconus and reduced QoL within the activity limitation domain.^7^ While indicating that HOAs contribute to reduced QoL in keratoconus, as total ocular HORMS were used for this analysis, further study is required to assess the impact of residual HOAs experienced during rigid lens wear on QoL outcomes.

Lifelong challenges for individuals with keratoconus mean they experience greater QoL impacts than would be predicted when considering visual acuity in isolation.^3^, ^8^, ^17^ The increased economic burden associated with specialised contact lenses is a significant contributing factor; this may include visits to practitioners with the necessary expertise to fit advanced contact lens designs, in addition to the financial outlay of the lenses themselves.^96^, ^97^ Other factors include challenges linked to comfort and suboptimal daily wearing time for corrective contact lenses,^3^, ^8^, ^57^, ^58^ bothersome symptoms (like ghosting) and ultimately the limitations imposed on the performance of routine activities, including driving in varied lighting conditions, household duties, use of digital devices or partaking in recreational activities.^3^, ^7^, ^8^, ^58^, ^94^, ^95^ With the emergence of ocular imaging technologies that provide improved understanding of corneal morphology,^98^ individuals with keratoconus will benefit from the development of clinical algorithms that predict optical performance for various vision correction modalities. A streamlined process for identifying where simpler lens modifications will suffice, or alternatively where more specialised solutions are required, could work to overcome some of the existing QoL challenges, including improving access to appropriate care for individuals with keratoconus.

## MANAGEMENT OF THE RESIDUAL OCULAR WAVEFRONT ERROR IN KERATOCONUS

Several strategies for the correction of residual ocular wavefront error have been attempted by researchers in efforts to mitigate reduced vision function and its impact on the QoL in individuals with keratoconus.^68^, ^99^ With varying degrees of success, these include the incorporation of additional specialised optical corrections onto soft contact lenses, corneal RGP lenses and scleral lenses.^99^ The latter has experienced increased interest in recent years due to the unique properties of this modality, making it well suited to the provision of wavefront‐guided corrections^12^ (Table 2).

**TABLE 2:** Management options for residual ocular wavefront error.

Modality	Modification	Description	Advantages	Disadvantages	Special considerations
Soft contact lenses	Base‐down prism	Base‐down prism commonly used to provide rotational stability for toric corrections Induces positive shifts in vertical coma; offsets negative vertical coma common in keratoconus	Reduced residual wavefront error through reduced magnitude negative vertical coma → improved vision Can be provided in addition to astigmatic correction	Reduced comfort with increased inferior thickness Lens location reliant on eyelid geometry and upright position Significant HOAs remain uncorrected in moderate to advanced disease	Simple addition to manufacture Must be provided in both eyes to prevent diplopia induced by differential vertical prism
	Increased thickness	Manufactured 0.3–0.6 mm thicker than standard^8^ Limits transfer of corneal irregularity through to contact lens surface	Reduced residual wavefront error through masking of anterior corneal irregularity → improved vision Can be provided in addition to aspheric and toric optics	Increased lens awareness with increased thickness Potential corneal hypoxia associated with thicker lenses Significant HOAs remain uncorrected in moderate to advanced disease	Commercially available lens designs like the KeraSoft IC® (kerasoftlens.com) utilise this parameter modification
	Posterior surface matching	Posterior lens surface specified to match the anterior corneal topography Offsets HOAs from anterior cornea	Reduced residual wavefront error through masking of anterior corneal irregularity → improved vision Improved rotational and translational stability	Residual HOAs due to no correction for posterior corneal irregularity Complex and costly manufacture	Not commercially available Would require additional front surface customisation to correct residual HOAs
	Wavefront‐guided corrections	Personalised front‐surface correction based on ocular wavefront measured over otherwise equivalent lens Corrections decentred for averaged translation and rotation during wear	Reduced residual wavefront error through correction of LOAs and significant reduction in HOAs → improved vision Good comfort Easy lens application and removal Practitioner familiarity with this modality	Specialised equipment required for lens fitting and manufacture High cost Lens rotation and translation lead to reduced vision Vision quality varies with pupil size Correction required for total residual ocular wavefront error (greater magnitude than residual HORMS in rigid contact lenses)	Reliant on alignment of wavefront‐guided correction with visual axis Partial correction may offer improved performance due to positional uncertainty HORMS vary with pupil size; pupil size should be considered when specifying wavefront‐guided corrections Vision performance consistent with corneal RGPs
Corneal RGPs	Base‐down prism	Base‐down prism commonly used to improve rotational stability for astigmatic corrections Induces negative shift in vertical coma; offsets positive residual vertical coma common in keratoconus	Reduced residual wavefront error through reduced magnitude positive vertical coma → improved vision Can be provided in addition to aspheric optic zones or front surface toric corrections for correction of residual astigmatism	Reduced comfort with increased inferior thickness Lens location reliant on eyelid geometry and upright position	Simple addition to manufacture Must be provided in both eyes to prevent diplopia induced by differential prism
	Aspheric optic zone	Front‐surface eccentricity applied to optic zone Spherical aberration induced offsets the residual positive spherical aberration common in keratoconus	Reduced residual wavefront error through reduced magnitude spherical aberration → improved vision	Lens translation → induced coma	Coma induced by decentration may result in worse image quality than for equivalent spherical design
Scleral contact lenses	Landing zone customisation	Toric, quadrant‐specific, and ‘free‐form’ landing zones Stabilise location limiting fluctuation in HOAs	Good comfort Improved centration Minimise rotation and translation Allows for reduced residual wavefront error through correction of residual astigmatism with front surface toric corrections Less fluctuation of residual wavefront error during wear	Increased practitioner skill required Specialised equipment required Accurate orientation required for lens application of quadrant and freeform peripheries	Improved stability allows for additional front‐surface optical corrections
	Corneal vault	Lens sagittal depth modified to control central corneal clearance	Reduced residual wavefront error as vault minimised → improved vision Reduced vault → reduced lens induced magnification	Low clearance risks corneal touch with lens settling or disease progression Significant changes in lens vault → alterations to required BVP	Positive shifts in spherical aberration from increased clearance may improve near vision quality Excessive clearance should be avoided to limit the potential for corneal hypoxia Should be kept consistent between eyes to minimise magnification differences
	BOZR	BOZR not constrained by ocular surface shape Modifications → changes in required lens BVP	Change in BVP → ↓/↑ FOZR → alterations to lens induced spherical aberration	BOZR significantly flatter than ocular surface may cause excessive clearance of peripheral optic zone BOZR significantly steeper than ocular surface may cause insufficient clearance of peripheral optic zone	Flatter BOZR can be used to improve clearance over low/decentred cones Alterations in required BVP can be used to avoid aniseikonia induced by anisometropia
	Aspheric optic zone	Front‐surface eccentricity applied to the optic zone Spherical aberration induced offsets the residual positive spherical aberration common in keratoconus	Reduced residual wavefront error through reduction in spherical aberration→ improved vision Minimal fluctuation compared to corneal RGPs due to rotational and translational stability	Offsetting residual positive spherical aberration results in reduced near vision performance	Need to compensate lens BVP for addition of negative spherical aberration Inferior decentration common to scleral lenses → induced coma providing offset of residual vertical coma
	Wavefront‐guided corrections	Personalised front‐surface correction based on ocular wavefront measured over otherwise equivalent lens Corrections decentred for alignment with the visual axis during wear	Lowest residual wavefront error of all modalities through correction of LOAs and HOAs→ improved vision Good lens stability minimises fluctuations in vision	Specialised equipment required for fitting and manufacture High cost Vision quality varies with pupil size	Residual ocular wavefront error will vary with pupil size; pupil size should be considered when specifying wavefront‐guided corrections

Abbreviations: BOZR, back optic zone radius; BVP, back vertex power; FOZR, front optic zone radius; HOAs, higher‐order aberrations; HORMS, higher‐order root‐mean‐squared error; LOAs, lower order aberrations; RGPs, rigid gas permeable contact lenses.

### Specialised soft contact lenses

Commonly used to provide rotational stability for toric corrections, incorporating base‐down prism into soft contact lenses has been demonstrated to induce positive shifts in vertical coma ($Z_{3}^{-1}$). With negligible impact on other HOAs, this effect increases in relation to the amount of prismatic power.^100^ Considering the dominance of negative vertical coma in keratoconus, this provides a partial reduction in HORMS. Although significantly less than the HORMS reduction afforded by rigid contact lenses, Jinabhai et al. reported vertical coma to improve from −0.77 ± 0.36 to −0.54 ± 0.41 μm, on average, for 22 participants with keratoconus when prism‐ballasted soft toric contact lenses were used.^101^

Customised soft contact lens designs for keratoconus are often manufactured 0.3–0.6 mm thicker than conventional lenses, providing a modest reduction in HORMS by limiting the transfer of the ocular surface irregularity through to their anterior surface.^8^ In addition to this, the KeraSoft IC® (Ultravision International Limited, kerasoftlens.com) utilises an aspheric front surface for correction of spherical aberration ($Z_{4}^{0}$), while incorporating spherical or prism ballasted toric corrections for LOAs. With a customisable periphery, up to two quadrants can be modified to allow better fitting on the asymmetric keratoconic corneal profile.^8^, ^10^, ^102^

In a retrospective study of 104 patients with keratoconus, Fernandez‐Velazquez compared outcomes for 94 eyes fitted with the KeraSoft IC® lens and 77 eyes fitted with Rose K2® (Menicon Co. Ltd., menicon.com), a keratoconus‐specific corneal RGP design. While there were no statistically significant differences in visual acuity between the two cohorts (0.04 ± 0.07 logMAR, *p* = 0.63), the researchers did not control for keratoconus severity.^102^ In a crossover study that included 27 individuals with keratoconus of varied severity, Kumar et al. compared visual acuity, contrast sensitivity, stereoacuity and residual HORMS between spectacle correction, KeraSoft IC®, Rose K2®, an alternative corneal RGP lens and a scleral lens. While performing better than spectacle correction, visual acuity and contrast sensitivity with the KeraSoft IC® were significantly worse than for all the rigid contact lens modalities, due to elevated HORMS.^51^ Devi et al. also reported reduced image quality for 15 members of this cohort using through‐focus computations based on the residual HOAs for each lens design.^103^

With respect to other soft contact lens designs, Chen et al. described significant improvements in rotational and translational stability compared to standard soft contact lens designs by implementing a posterior surface customised to match anterior corneal shape in three eyes with moderate keratoconus. Although these lenses provided significantly reduced HORMS by correcting anterior corneal aberrations in a manner analogous to the tear lens of a corneal RGP lens, using conventional front‐surface optics failed to compensate for the contribution of aberrations from the posterior cornea or crystalline lens.^104^

### Corneal RGP contact lenses

Although the observed positive shift in vertical coma induced by base‐down prism in soft contact lenses can be beneficial in keratoconus,^100^ this change would be undesirable for rigid contact lens correction where the residual vertical coma is already positive in sign.^11^, ^70^ To examine this, Carballo‐Alvarez et al. compared the difference in ocular HOAs with two lenses: a corneal RGP lens with spherical optics and an otherwise identical lens with the addition of 1.6Δ base‐down prism. In a cohort of 17 eyes with mild to moderate keratoconus, inclusion of the base‐down prism provided a greater reduction in HORMS, including a lower magnitude of positive vertical coma.^15^ In contrast to soft contact lenses,^100^, ^101^ the addition of prism in rigid contact lenses induced a negative shift in vertical coma.^15^

The unique features of each modality explain why base‐down prism induced opposing changes in vertical coma while remaining beneficial. For both soft and rigid contact lenses, the addition of base‐down prism led to increased lens thickness inferiorly.^9^, ^100^ When placed onto the cornea, the prescribed contour of a rigid contact lens is maintained, manifesting as a forward rotation of the optic zone inferiorly. The increased optical pathlength relative to the superior optic zone induces a negative shift in vertical coma ($Z_{3}^{-1}$),^15^ thus offsetting the opposing residual aberration. Meanwhile, the positive shift seen with soft contact lenses is likely a result of the lens conforming to the ocular surface, with increased inferior thickness providing additional masking of the irregular inferior cornea.

### Scleral contact lenses

**FIGURE 2:**
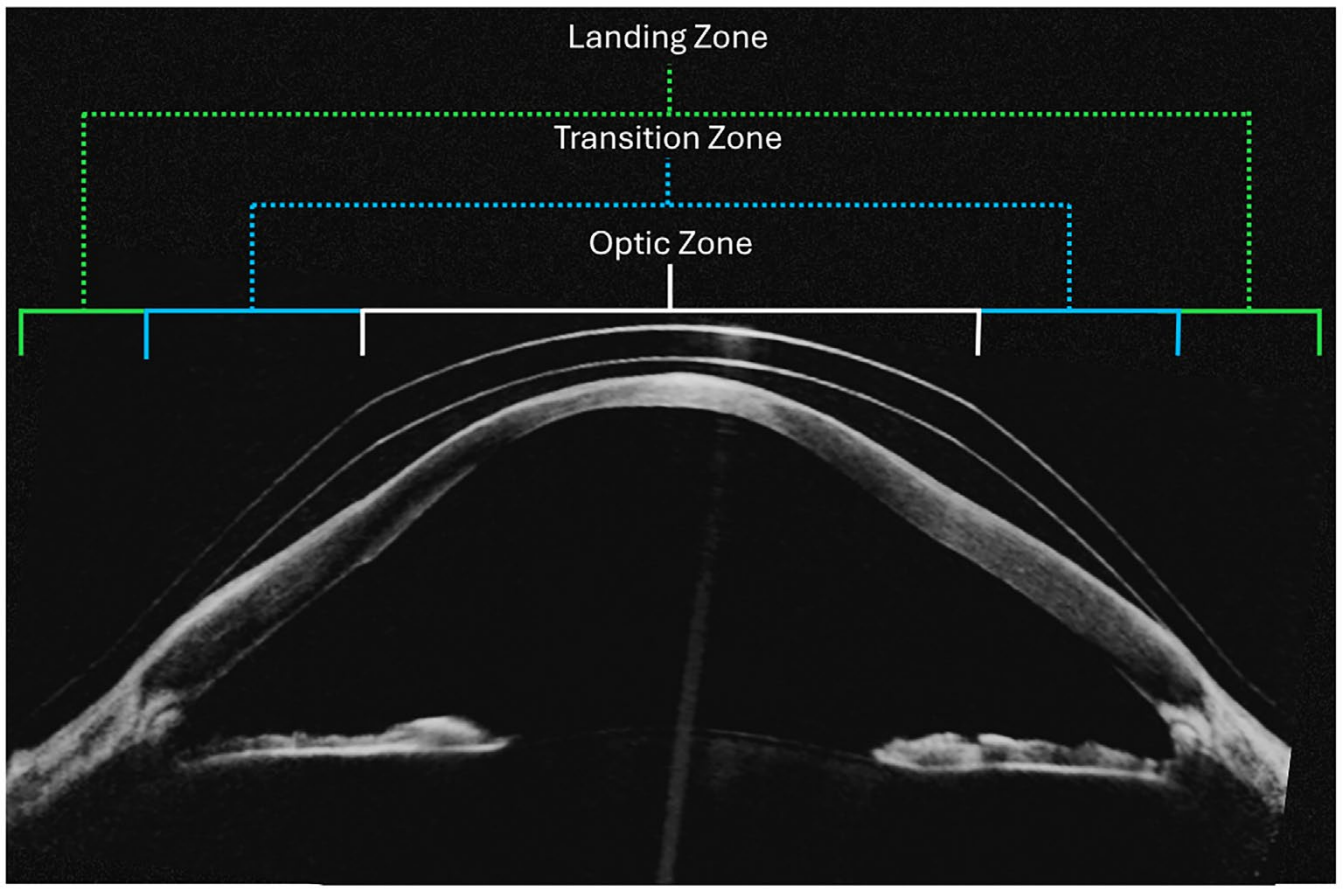
OCT B‐scan of a scleral lens in situ on an eye with advanced keratoconus, demonstrating the formation of the tear lens between the back surface of the lens and the ocular surface. Annotations delineate the three zones of the scleral lens; Optic Zone (white), Transition Zone (blue) and Landing Zone (green).

Scleral lenses offer many advantages over corneal RGP lenses and specialised soft contact lenses for reducing HORMS and improving vision quality in individuals with keratoconus,^13^, ^49^, ^105^, ^106^ thereby demonstrating the capacity for superior high‐ and low‐contrast VA.^49^ Manufactured from RGP materials, scleral lenses are larger in diameter than corneal lenses and are designed to vault the cornea entirely; the weight of the lens is borne beyond the limbus.^1^, ^8^, ^12^, ^13^, ^72^, ^105^, ^107^, ^108^ (Figure 2) Although scleral lens designs vary in their individual construction and may involve the use of proprietary terminology for specific lens design features, the recommended Scleral Lens Education Society nomenclature defines three main zones to the lens design: the optic zone, the transition zone and the landing (or haptic) zone.^12^, ^105^, ^107^ Parameter modifications in each zone can influence the composition of the ocular wavefront and image quality.

Referred to as the optic zone, the central optical portion of the scleral lens is defined by the BOZR and FOZR parameters. However, unlike corneal RGP lenses, the ocular surface ‘vault’ means the BOZR is not required to align with the anterior corneal contour.^12^, ^13^, ^74^ The outermost zone, designated as the landing zone, comes to rest on the bulbar conjunctiva, overlying the sclera. Given the reduced sensory innervation in this region, scleral lenses generally provide improved comfort over corneal RGPs.^13^, ^47^, ^49^, ^109^ The parameters of the landing zone are specified to align with the scleral contour, with rotationally symmetric, toric, quadrant‐specific and ‘free‐form’ landing zones being available.^13^, ^47^, ^57^, ^59^, ^60^, ^105^, ^109^, ^110^ Bridging the gap between the optic zone and landing zone is the transition zone, with parameter modifications primarily used to control the sagittal depth of the scleral lens to ensure complete corneal clearance.^12^, ^13^, ^105^

Scleral lenses are applied to the eye while filled with preservative‐free fluid,^12^, ^57^, ^59^ with circumferential alignment of the landing zone on the ocular surface ensuring a portion of the fluid is retained at the exclusion of air. Analogous to the tear lens for corneal RGP lenses, the fluid reservoir or fluid lens within the optic zone neutralises around 90% of the anterior corneal refractive power, including HOAs. The scleral lens BOZR determines the BVP of this fluid lens, with additional refractive correction achieved through modifications to the FOZR.^13^, ^47^, ^72^, ^74^, ^76^, ^105^

The broad haptic of scleral lenses can be customised to provide an even landing on the asymmetric sclera, with a goal to achieve an even distribution of lens bearing. This affords improved stability and can minimise HOAs induced by lens translation and rotation, or lens flexure.^78^ A slight inferior temporal lens decentration is commonly observed with scleral lenses; on average, 0.1–1.0 mm horizontally and 0.2–1.7 mm vertically.^12^ This can be varied with alterations to sagittal depth and landing zone construction, with improved performance achieved by increasing customisation,^12^, ^60^, ^75^ stabilising rotation to as little as 1° and translation to less than 0.1 mm in either direction.^60^

Yildiz et al. reported a reduced accommodative response for participants in scleral lens wear compared to those using corneal RGP lenses, theorising the influence of microstructural changes to the posterior chamber induced by pressure from the scleral lens landing zone.^47^ Both groups had similar average residual spherical aberration ($Z_{4}^{0}$),^47^ ruling this out as the source of discrepancy. As rotationally symmetric scleral lenses were used, alternative landing zone constructions with improved distribution of bearing forces warrant further investigation.

The excellent rotational and translational stability of scleral lenses, coupled with a wide optic zone that is unconstrained by ocular surface curvature, provides the opportunity to apply additional optical corrections for enhanced vision quality.^8^, ^10^ Rotational stability allows for the use of front‐surface toric corrections when significant residual astigmatism is present.^13^ Vaulting of the cornea permits adjustments to the BOZR, independent of keratometry values, without disruption to lens stability. Substantial alterations to the fluid lens can be utilised to modify the required scleral lens BVP; this is particularly useful for the correction of high ametropia, to minimise spherical aberration ($Z_{4}^{0}$) or to adjust magnification in the case of anisometropia.^74^

Although complete ocular surface clearance is essential during scleral lens wear, an excessive vault is contraindicated. Central corneal clearance of 200–300 μm is recommended to limit hypoxia‐induced corneal oedema,^12^, ^105^ while avoiding mechanical changes to the ocular surface from lens settling.^111^, ^112^ Optical implications should also be considered when deciding on an appropriate extent of lens clearance. With increases generating greater magnification, care should be taken to ensure inter‐ocular differences in clearance are minimised.^74^ In addition, increasing scleral lens vault by 100 μm has been demonstrated to modify the fluid lens BVP by as much as +0.12 to +0.50 D,^74^, ^105^, ^113^ depending on the BOZR and its relationship with the anterior corneal shape.^74^

Residual HORMS are also influenced by modifications to the scleral lens vault, with Villa et al. observing elevated coma ($Z_{3}^{-1}$, $Z_{3}^{1}$), spherical aberration ($Z_{4}^{0}$) and trefoil ($Z_{3}^{-3}$, $Z_{3}^{3}$) associated with increased lens clearance.^105^ While Balakrishnan et al. found no significant change to HORMS as the lens vault varied, visual acuity was significantly better with the lowest lens clearance.^76^ The BOZR and BVP of the test scleral lenses used in both studies were not held constant as corneal clearance was varied, meaning results should be interpreted with caution. Using computational raytracing simulations, Piñero et al. confirmed that distance image quality is optimised when scleral lens clearance is minimised; however, positive shifts in spherical aberration ($Z_{4}^{0}$) associated with increased clearance provided improved image quality for near tasks.^113^ Although this has implications in regard to presbyopia, near spectacle corrections can be worn if best distance clarity is desired.

Specification of front‐surface eccentricities greater than zero translates to a progressive reduction of surface curvature away from the centre of the optic zone. The negative spherical aberration ($Z_{4}^{0}$) that is induced, in proportion to eccentricity, can be used to offset residual spherical aberration ($Z_{4}^{0}$), which is often positive. Gumas et al. reported reduced spherical aberration ($Z_{4}^{0}$) with the use of aspheric optic zones in scleral lenses. Although no significant improvement in visual acuity was observed,^25^ all measurements were collected in a single session allowing no time for neural adaptation. Balakrishnan et al. also demonstrated improved image quality when front surface asphericity was applied to scleral lenses in a cohort of individuals with mostly moderate and advanced keratoconus. When compared to an equivalent spherical lens, scleral lenses with a 0.30 front surface eccentricity provided shifts in spherical aberration ($Z_{4}^{0}$) from 0.12 ± 0.14 to −0.01 ± 0.07 μm, and total coma ($Z_{3}^{-1}$, $Z_{3}^{1}$) from 0.57 ± 0.34 to 0.29 ± 0.15 μm.^72^ As coma ($Z_{3}^{-1}$, $Z_{3}^{1}$) is induced by decentration of aspheric surfaces,^9^, ^29^, ^73^, ^74^ the inferior temporal decentration typically observed with scleral lenses^49^ was likely responsible for the additional offset of this residual term.

### Wavefront‐guided contact lens corrections

Regardless of modality, wavefront‐guided contact lenses incorporate a corrective optical ‘patch’ that generates the inverse of the ocular wavefront measured over an otherwise equivalent lens.^78^ The goal is to improve vision function via HORMS reduction.^32^, ^33^, ^37^, ^114^ The fitting approach for wavefront‐guided lenses involves performing aberrometry over a contact lens corrected for LOAs while controlling the patient's fixation to ensure measurements are conjugate with the visual axis; this allows for the residual ocular wavefront to be determined.^66^, ^71^, ^78^, ^115^ The RMS of the component HOAs is scaled to a predetermined pupil size, and a lens is subsequently manufactured to incorporate this optical correction.^78^, ^99^ Although ocular HOAs remain dynamic, the fluctuations associated with the tear film and accommodation are of relatively low significance in keratoconus, considering their scale compared to residual HORMS.^14^, ^47^

When deciding on an appropriate contact lens modality to carry wavefront‐guided corrections, lens fitting challenges including decentration, rotation and translation need to be considered in the context of the magnitude of the residual aberrations. A misalignment between the centre of the corrective patch and the visual axis will result in reduced vision quality, worsened with both increasing deviation and the magnitude of residual HOAs targeted for correction.^41^, ^60^, ^110^, ^115^ To limit potentially deleterious impacts on visual acuity, some authors have advocated for partial correction of the HOAs, targeting specific Zernike terms to flatten the wavefront in the region surrounding the visual axis.^15^, ^29^, ^37^, ^60^, ^114^

Partial correction of residual HOAs was investigated by Shi et al. based on the average rotation and translation of a well‐fitted scleral lens in three individuals with keratoconus. The authors determined residual HOAs and applied a stochastic parallel gradient descent methodology to determine the partial wavefront‐guided correction that provided the best logVSX while accounting for location variance. Considering the changes in lens position during the course of wear, compared to full correction, this provided an improvement of 0.14 logMAR (seven additional letters),^71^ demonstrating the importance of weighing the benefits offered by a complete correction of residual HOAs against the negative impacts if new HOAs are induced as a result of misalignment of the lens on‐eye.

#### Wavefront‐guided soft contact lenses

With a total diameter larger than the horizontal visible iris diameter (HVID), soft contact lenses are influenced by asymmetry of the corneoscleral profile, resulting in an average 0.4 mm temporal displacement relative to the pupil centre.^9^, ^99^ Although more stable than corneal RGP lenses with respect to lens rotation and translation, soft contact lenses require sufficient on‐eye mobility to support optimal corneal physiology,^116^ leading to potential challenges for the provision of wavefront‐guided soft lens corrections.

To assess the change in image quality created by standard misalignments of a wavefront‐guided soft contact lens, de Brabander et al. calculated the change in area under the MTF created by translations and rotations of the magnitude typically experienced during soft contact lens wear. They concluded that on‐eye lens translations should be no more than 0.5 mm and rotations less than 5° for appropriate vision benefit.^117^ In a similar study, Jinabhai et al. simulated the HOAs induced by rotation of a wavefront‐guided soft contact lens correction. Although in agreement for rotation, they concluded that on‐eye vertical lens translation should be no more than 0.1 mm to maintain an acceptable vision correction.^41^ However, given that HORMS is a poor predictor of vision quality, this finding may overstate the impact of higher frequency HOA terms like trefoil ($Z_{3}^{-3}$, $Z_{3}^{3}$).

Examining the benefit of improved on‐eye lens positioning for wavefront‐guided corrections in soft contact lenses, Sabesan et al. assessed customised corrections with lens location adjusted to account for the translation observed when an equivalent conventional prism‐ballasted lens was worn. Among a sample that included one moderate keratoconic and two eyes with advanced keratoconus, these lenses proved effective in reducing vertical coma ($Z_{3}^{-1}$). However, this was not the case for trefoil ($Z_{3}^{-3}$, $Z_{3}^{3}$) thereby limiting the total HORMS reduction. While the average HORMS was reduced to 0.93 ± 0.19 μm with the wavefront‐guided lens, compared with 2.75 ± 0.90 μm in the conventional lens, this was still nearly twice that for non‐diseased eyes. Despite this incomplete correction of HORMS, an average improvement of 2.1 lines in high‐ and low‐contrast visual acuity was observed,^118^ highlighting the benefit of partial HORMS reduction.

In a proof‐of‐concept study, Marsack et al. tested an alternative wavefront‐guided soft contact lens on one individual with moderate keratoconus. Compared to their habitual soft lens (SofLens 66; Bausch and Lomb, bausch.com), they experienced an improvement of 1.5 lines for high‐contrast and 1.0 line for low‐contrast VA.^119^ While HORMS was reduced from 0.77 μm in the habitual lens to 0.386 μm in the wavefront‐guided lens,^119^ this comparison is limited due to the otherwise vast differences between the lens designs.

#### Wavefront‐guided scleral lenses

In addition to the improved optical performance afforded by individual modifications of scleral lens parameters, on‐eye stability makes this lens modality ideal for wavefront‐guided refractive corrections.^8^, ^12^, ^13^, ^66^, ^71^, ^74^, ^77^, ^99^, ^110^, ^120^ Inventory from a scleral lens fitting set is unlikely to match the final (required) lens parameters, as related to factoring in the lens BVP, eccentricity, decentration, rotation and tilt. All these attributes will influence HOAs, making it inappropriate to use trial lenses to determine wavefront‐guided scleral lens corrections. Instead, this must be performed with an optimally fitted lens.^78^ Significant customisation (beyond that for scleral lenses with standard optics) is often necessary to achieve acceptable lens stability, requiring substantial practitioner expertise and taking on average 2.6 lens changes over 3.8 visits, although up to 16 iterations have been reported.^109^ Once a suitable physical lens fitting is established, lens translation and rotation are quantified relative to the visual axis, with a duplicate lens made to incorporate the wavefront‐guided correction, offset to account for the appropriate position of the lens during wear.^60^, ^66^, ^71^, ^77^, ^78^, ^110^, ^121^, ^122^ With multiple lens iterations and increased chair‐time,^109^ this process can incur significantly higher costs for end users compared to standard scleral lens corrections.^13^, ^59^, ^77^

Using a similar approach, Sabesan et al. fitted wavefront‐guided lenses to 11 eyes with advanced keratoconus. Residual HORMS and measures of visual function were recorded and compared to an equivalent scleral lens with standard optics. The wavefront‐guided lens reduced HORMS magnitude by 3.1 times to an average of 0.37 ± 0.19 μm for a 6 mm pupil. High‐contrast logMAR VA was improved by approximately two lines, and contrast sensitivity was 2.4, 1.8 and 1.4 times better for spatial frequencies of 4 cycles per degree, 8 cycles per degree and 12 cycles per degree, respectively. Although HORMS improved to levels approaching those observed in non‐diseased eyes, VA did not demonstrate an equivalent enhancement. This discrepancy is likely due to VA measurements being obtained during the same visit as lens dispensing, without sufficient time to allow for neural adaptation to the enhanced image quality.^121^

In a similar study, Marsack et al. examined 14 eyes with moderate to severe keratoconus, comparing an aspheric front‐surface scleral lens with spherical equivalent correction to a duplicate lens incorporating a front‐surface, wavefront‐guided correction.^122^ In line with the findings of Sabesan et al.,^121^ significant improvement in HORMS was observed, with 10 of the 14 eyes reaching within one standard deviation of age‐matched norms, and an average improvement of 1.5 lines in logMAR visual acuity, from +0.14 to −0.01. Inconsistent on‐eye lens stability resulted in increased HORMS for three eyes,^122^ highlighting the importance of establishing lens stability prior to wavefront‐guided corrections.^60^ Vision quality metrics including logVSX were calculated for the 10 eyes demonstrating a reduction in HORMS, with values indicating further improvements in vision function likely if time were allowed for adaptation.^122^

The mismatch observed between reduced HORMS and only modest improvements in vision performance with wavefront‐guided corrections^121^, ^122^ may result when functional parameters are quantified when the lens is dispensed (rather than at a review visit), as this will not account for potential altered neural adaptation with lens wear over time.^39^ Addressing this, Hastings et al. conducted a randomised crossover study comparing outcomes for front‐surface aspheric scleral lenses and wavefront‐guided scleral lenses following 8 weeks of lens wear. Compared to participants' habitual corrections, HORMS was found to improve by 48% with the aspheric scleral lens and 71% with the wavefront‐guided scleral lens; the latter provided improvement to (on average) 0.26 ± 0.077 μm HORMS. As such, 85% of participants reached age‐matched normal HORMS levels, compared to 40% with the aspheric scleral lens. Following habituation, 85% of participants achieved visual acuities equivalent to age‐matched normative values with the wavefront‐guided scleral lenses, compared to 50% for the aspheric scleral lens; a similar trend was observed for contrast sensitivity. As predicted, when individuals were afforded time to habituate to wavefront‐guided vision corrections, significant improvements in vision function align with the (objective) reduction in HORMS.^39^

Gelles et al. recently reported interim findings from a crossover‐controlled clinical trial comparing outcomes after 1‐month habituation with a commercially available wavefront‐guided scleral lens and the same scleral lens without wavefront‐guided optics. Evaluating the translation of wavefront‐guided corrections to the clinical setting, 31 eyes with irregular corneal astigmatism of varied origin, including keratoconus, post‐refractive ectasia and penetrating keratoplasty were assessed, without excluding those with corneal or lenticular opacity. Compared to standard scleral lenses, the wavefront‐guided corrections provided an average additional HORMS reduction of 56% for a 5 mm pupil, to 0.29 ± 0.18 μm. Following habituation, visual acuity was, on average, 0.12 ± 0.11 logMAR better with the wavefront‐guided lens, improving to 0.03 ± 0.11.^78^ This study demonstrates the potential to use commercially available wavefront‐guided lenses to enhance visual performance, regardless of corneal disease severity.

## CONCLUSION

Although conventional corneal RGP contact lenses offer a range of benefits, including with respect to vision quality, compared to spectacles and standard soft contact lenses for keratoconus, HORMS remain elevated compared with non‐diseased eyes. While the tear lens, formed when a rigid contact lens is placed on the eye, neutralises the bulk of the HOAs arising from the anterior cornea, it has no influence on the posterior cornea and thus any irregularity here remains uncorrected. As the main source of residual HOAs, posterior corneal irregularity increases with disease severity in keratoconus. Elevated HOAs, like vertical coma ($Z_{3}^{-1}$) and spherical aberration ($Z_{4}^{0}$), result in reduced vision quality and disruptive subjective symptoms (like ghosting), with activity limitations reducing QoL despite best conventional refractive correction.

Various parameter modifications to contact lenses may be employed to minimise residual uncorrected wavefront error, offering improvements in vision quality for individuals with keratoconus. The addition of aspheric optics, base‐down prism and increased thickness, to mask some of the irregularity of the anterior cornea, can improve vision quality with soft contact lenses. Parameter adjustments that improve the centration of a corneal RGP lens will limit coma induced by lens decentration. Adding front‐surface asphericity to a corneal RGP lens can offset residual spherical aberration ($Z_{4}^{0}$); however, this should be applied with caution as decentration and translation will result in increased coma. Other simple additions like the incorporation of base‐down prism may be of benefit, both stabilising rotation while reducing manifest vertical coma.

The unique properties of scleral lenses allow for a range of additional parameter adjustments that can moderate residual HOAs. Improved rotational and translational lens stability reduce the likelihood of inducing unwanted HOAs from unexpected on‐eye lens misalignment, with aspheric front‐surface optics effective in this setting. Unlike corneal RGP contact lenses, which have parameters defined by the patient's corneal profile, modifications to the BOZR and lens sagittal depth can be used to modify spherical aberration ($Z_{4}^{0}$) for scleral lenses. In addition, alterations to the lens optic zone tilt may prove useful for reducing coma, although further investigation is required.

Wavefront‐guided contact lenses are an emerging technology that offers the capacity for additional HORMS reduction, to impart significant improvements in vision quality. Requiring precise correspondence between the lens (on‐eye) and the visual axis, modalities that have high amounts of lens translation and rotation are unsuitable for these corrections. Although soft contact lenses have improved stability compared to corneal RGP lenses, the required amount of HORMS correction is higher due to negligible masking of anterior corneal irregularity, thereby amplifying the negative impacts of lens decentration and rotation. In contrast, appropriately fitted scleral lenses tend to show predictable stability, combined with lower residual HORMS, making them the ideal vehicle to incorporate wavefront‐guided corrections.

With increased cost, complexity and clinical chair‐time required to prescribe wavefront‐guided scleral lenses, clinicians should weigh the potential benefits against simpler modifications to the lens design for each clinical presentation. Considering that visual demands are task dependent, neural adaptation in the setting of chronically reduced image quality may render the habitual correction acceptable. Alternatively, other patients may prefer wavefront‐guided corrections, particularly in scotopic conditions where HORMS are elevated due to enlarged pupils. When wavefront‐guided corrections are to be provided, this should be based on aberrometry and performed over an optimally fitted contact lens. Although further research is required to ascertain the time course of neural adaptation, sufficient time should be afforded prior to determining improvements in visual function.

A vast array of specialised contact lens options is now available for vision correction in keratoconus; however, these are not ‘one size fits all’. Instead, the appropriate choice needs to be tailored to meet the needs of the individual presentation. In many cases, standard keratoconus‐specific corneal RGPs with parameters determined from a trial lens fitting can provide acceptable vision and comfort. Often, the determination is more nuanced than this, with consideration for corneal parameters, lifestyle factors and vision requirements.

In the absence of the necessary tools to measure residual wavefront error, clinicians are likely unaware of the visual compromise that their patients may be tolerating. With the increasing availability of instruments incorporating wavefront aberrometry, this clinical measure should ideally become routine in keratoconus management. Looking forward, this determination, coupled with enhanced understanding of individuals' corneal morphology, as provided by advancements in corneal imaging, may assist clinicians in identifying where wavefront‐guided lenses will provide benefit for patients, beyond those offered by simpler lens modifications.

## AUTHOR CONTRIBUTIONS

**Gavin Swartz:** Conceptualization (lead); methodology (lead); project administration (lead); writing – original draft (lead); writing – review and editing (lead). **Khyber Alam:** Project administration (equal); supervision (equal); writing – review and editing (equal). **Alex Gentle:** Project administration (equal); supervision (equal); writing – review and editing (equal). **Laura E. Downie:** Project administration (equal); supervision (equal); writing – review and editing (equal).

## FUNDING INFORMATION

None.

## CONFLICT OF INTEREST STATEMENT

G. Swartz, K. Alam, A. Gentle, L. E. Downie declare no conflict of interest.
